# Estimating the catchment population and incidence of severe acute respiratory infections in a district hospital in Boussé, Burkina Faso

**DOI:** 10.7189/jogh.10.010422

**Published:** 2020-06

**Authors:** Jennifer L Milucky, Toussaint Compaore, Franck Obulbiga, Gretchen Cowman, Cynthia G Whitney, Brice Bicaba

**Affiliations:** 1 Centers for Disease Control and Prevention, National Center for Immunization and Respiratory Diseases, Division of Bacterial Diseases, Atlanta, Georgia, USA; 2 Ministry of Health, Directorate for the Protection of Population Health, Ouagadougou, Burkina Faso; 3 Centers for Disease Control and Prevention, Center for Global Health, Ouagadougou, Burkina Faso

## Abstract

**Background:**

The primary cause of death in Burkina Faso is lower respiratory tract infections, accounting for 1 in 7 deaths. The Ministry of Health is building surveillance for severe acute respiratory infections (SARI) in four districts. This study sought to determine the catchment area of the Boussé district hospital and to describe disease burden of individuals hospitalized for SARI.

**Methods:**

Data were collected from hospital log books to identify individuals with a SARI diagnosis during 2015 and 2016. Residence of SARI patients was recorded to determine the catchment area of the hospital. Population data were used to estimate SARI incidence rates.

**Results:**

Investigators reviewed logs for 3034 hospital admissions; 885 SARI cases were identified. Five communes were identified as the hospital catchment area, with 770 SARI patients residing in these communes. The SARI incidence rate (IR) for all ages was 136 (95% confidence interval (CI) = 115, 161) and 266 (95% CI = 236, 300) cases per 100 000 population for 2015 and 2016, respectively. Children <1 (RI = 1111 cases per 100 000, 95% CI = 1047, 1178, and RI = 2425 cases per 100 000, 95% CI = 2330, 2524) and adults ≥65 years old (RI = 377 cases per 100 000, 95% CI = 341, 417, and RI = 816 cases per 100 000, 95% CI = 762, 874) had the highest burden of disease for 2015 and 2016, respectively.

**Conclusion:**

Our analysis found high rates of SARI, especially among children <1 year of age, and marked variation in incidence between the years studied. These baseline data and the method developed will be useful for the new SARI surveillance system.

Lower respiratory tract infections (LRTIs) caused an estimated 2.7 million deaths worldwide in 2015, with more than 700 000 of those deaths occurring in children less than 5 years of age [1]. Among children under 5 years of age, 15% of deaths are attributed to acute respiratory infections (ARIs), and over 40 percent of ARI-associated deaths in children occur in Africa [2]. Burden of ARIs is highest among children less than 5 years of age and in adults older than 65 years of age [3-6]. Despite its morbidity and mortality, the epidemiology and causes of LRTIs, particularly in Africa, are not well described [7]. Additionally, much of the existing data does not come from population based surveillance, limiting the ability to report disease incidence.

Burkina Faso is a land-locked country situated in sub-Saharan West Africa with a population of approximately 18 million people in 2016 [8]. The primary cause of death in Burkina Faso is LRTIs, which account for 14.3% of all deaths [9]. The Ministry of Health in Burkina Faso recently started surveillance for severe acute respiratory infections (SARI) using the World Health Organization’s (WHO) SARI case definition [10] as part of a family of efforts to strengthen infrastructure for Global Health Security. Four district-level hospitals in Bogodogo, Boussé, Hounde, and Kongoussi are participating in the surveillance.

Currently, limited data are available for both the number of people with SARI who are treated at district hospitals and the population of individuals who seek care at these hospitals for respiratory infections. To determine the incidence of SARI and enable comparisons with other populations, an estimate of the “at risk” population, also known as the catchment population, is needed for the sentinel hospitals. One approach for obtaining an estimate of this denominator is based on the WHO method to estimate the disease burden associated with seasonal influenza in a sentinel hospital catchment area [11]. Knowledge of a health care facility’s catchment area is important for assessing health service utilization and calculating incidence rates of disease.

As the Ministry of Health was implementing SARI surveillance in Burkina Faso, we used the WHO method to estimate the catchment population and calculate SARI incidence for one of the participating district hospitals. The objectives of this study were: 1) to estimate the geographic catchment area and population for the Boussé district hospital during the period of 1 January 2015 to 31 December 2016, and 2) to quantitatively describe disease burden of ARIs requiring hospitalization in the Boussé district hospital catchment area using Boussé district hospital log books.

## METHODS

The geographic scope of this study was the health district of Boussé, located in the Plateau-Central region of Burkina Faso. Boussé Centre Medicale avec Antennae (CMA) is a district-level hospital within the Boussé health district. Data were retrospectively collected from hospitalization log books where the chief medical officer indicated any SARI patients might be hospitalized (the maternity, pediatric, and emergency wards) at Boussé CMA. Available logs were reviewed for all patients admitted for hospitalization during the period of 1 January 2015 to 31 December 2016. All patient information was entered into MS Excel (Microsoft Inc, Seattle WA, USA).

Admission and discharge diagnoses listed on the logs were used to identify patients who would likely meet the WHO SARI criteria [10]. To determine which admission and discharge diagnoses to include, we started with the list of several possible ARI diagnoses in the WHO method [11]. During data collection, any additional diagnoses identified within Boussé CMA hospitalization log books considered to be ARIs by local clinical staff were added to the initial list. Information was recorded for patients with any of these diagnoses. Admission and discharge diagnoses for suspected SARI cases were then categorized into diagnosis groups: pneumonia, bronchitis, bronchiolitis, asthma, tuberculosis, or other respiratory disease. To be included in the analysis as a suspected SARI case, an illness had to meet the following two criteria: 1) the patient with the illness must have received at least one acute respiratory infection diagnosis upon admission and/or at discharge, and 2) the patient must have been hospitalized at Boussé CMA between 1 January 2015 to 31 December 2016.

Each case was assigned a unique ID. The commune where the patient resided was recorded to determine where the population served by Boussé CMA resides. Patient name and residence were used to identify patients with more than one suspected SARI admission during a 30-day period at the Boussé CMA; only the first of multiple admissions within 30 days were counted in the analysis as subsequent admissions were considered to be either a continuation of the original illness or a hospital acquired infection. Identifying information was removed once data collection was completed. Additional demographic information, other non-SARI diagnoses, and hospital outcome data were also collected.

**Figure 1:**
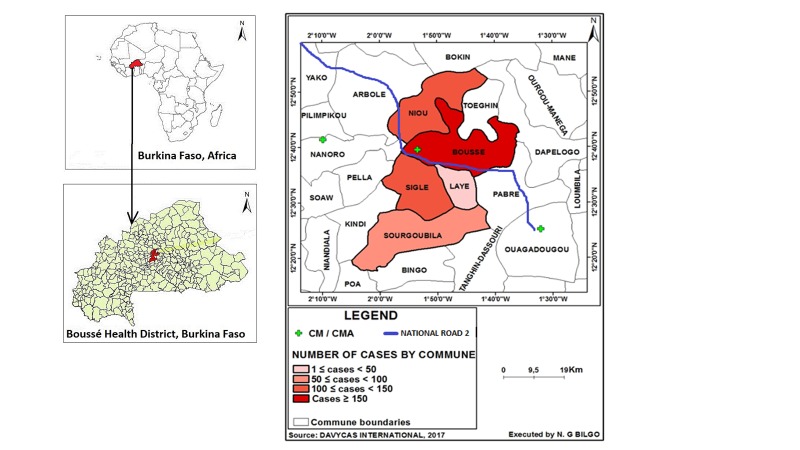
Map representing Boussé district hospital suspect severe acute respiratory infection (SARI) cases by commune from 1 January 2015 to 31 December 2016 (n = 781). Bousse CMA SARI catchment area defined as communes of Bousse, Niou, Sigle, Sourgoubila, and Laye (n = 667) from districts of Bousse and Nanoro. CMA – Centre Medical avec Antennae, CM – Centre Medical.

Based on the method described in the WHO’s *A Manual for Estimating Disease Burden Associated with Seasonal Influenza* [11], communes were rank-ordered according to the number of cases residing in each commune. The Boussé CMA geographical catchment area was then defined as the communes where approximately 85% of the patients admitted with acute respiratory infections resided, following WHO methods [11]. Patients were excluded from the catchment area determination if their commune level residence data were missing. Suspected cases from these communes were mapped using ArcGIS to visualize geographical dispersion and outliers (**Figure 1**). Expert opinion from the Boussé CMA epidemiologist was sought to identify other health facilities within Burkina Faso that were likely to admit at least 10% as many patients with suspected SARI as those admitted to Boussé CMA among people residing in the Boussé district CMA’s geographical catchment area.

After the communes comprising the catchment area for the Boussé CMA were identified, commune population projection data from census district records [12] were used to determine the catchment population of the Boussé CMA from 2015 and 2016. Suspected SARI cases lacking commune level data were not included in the determination of the SARI catchment area, but 85% of these cases were randomly selected and included in incidence calculations based on the assumption that the residence distribution of these suspected cases was similar to that of suspected cases with recorded commune level data. Descriptive analyses were conducted to describe basic demographics of SARI patients and to determine the frequency of admission and discharge diagnoses recorded in the hospital log book. Single imputation was done to account for seven weeks of missing data from the pediatric ward in 2015; the ratio of cases for each epi week of 2015 to 2016 was calculated and the overall mean ratio multiplied by the 2016 weekly case counts applied for the seven weeks in 2015 with missing data. The 1st epi week of 2015 began with Sunday, 4 January through Saturday, 10 January. SARI incidence rates (cases per 100 000 population) and 95% confidence intervals for SARI overall and by age group were calculated for the catchment area assuming a Poisson distribution. Data analyses were conducted using SAS 9.4 (SAS Institute, Inc., Cary, NC, USA). This study was considered part of surveillance activities and did not require ethics review.

## RESULTS

**Figure 2:**
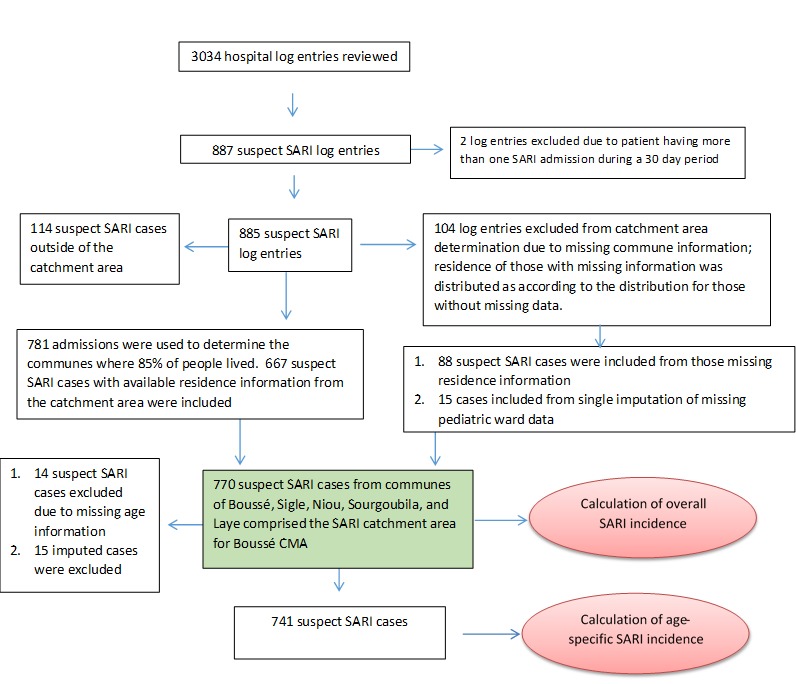
Data collection flow. SARI – severe acute respiratory infection, CMA – Centre Medicale avec Antennae.

Logs were available for review for 3034 hospital admissions occurring during 1 January 2015 to 31 December 2016 (**Figure 2**). Log books were unavailable for the pediatric ward for epi weeks 1-5 in January and February 2015 and epi weeks 43 and 44 during October and November 2015; six months of hospital logs were missing for the maternity ward in both 2015 and 2016. However, only five cases overall were identified from the maternity ward logs for months where data were available. Using single imputation, an additional 15 cases were added to 2015 to account for the missing weeks of pediatric data.

### Determination of catchment area and population

Among the 3034 admissions noted on hospital logs, 885 suspected SARI cases from the Boussé CMA were identified after excluding two admissions that were considered continuations of the original illness or suspected hospital acquired infections. Of these, 104 patients with missing residence information were not included in determination of the catchment area (**Figure 2**). The remaining 781 admissions were rank ordered by commune of residence (**Table 1**). The communes of Boussé, Noiu, Sigle, Sourgoubila, and Laye constituted 677 (85.4%) of the cumulative number of suspect SARI cases identified. The population of the communes comprising the Boussé CMA catchment area consisted of 188 741 persons in 2015 and 193 409 persons in 2016 [12]. The largest proportion (42%) of suspected SARI cases were from the commune of Boussé. There are no other hospitals within these five communes, and, according to the local epidemiologist, there are no other hospitals just outside these communes that receive a high number of patients from these communes. After assuming that those with missing residence information (n = 104) were distributed the same way as those with information (85.4% from the 5 communes) and including 15 imputed cases from missing data in the pediatric ward, a total of 770 SARI cases were estimated to occur among catchment area residents during the two years of observation.

**Table 1:** Residence of patients seeking care at Boussé district hospital for severe acute respiratory infections (SARI), by commune

Residence by commune*	N = 781†	%	Cumulative %
**Boussé**	329	42.1%	**42.1%**
**Niou**	137	17.5%	**59.7%**
**Sigle**	109	14.0%	**73.6%**
**Sourgoubila**	55	7.0%	**80.7%**
**Laye**	37	4.7%	**85.4%**
Pella	30	3.8%	89.2%
Arbole	25	3.2%	92.5%
Toeghin	24	3.1%	95.5%
Nanoro	13	1.7%	97.2%
Ouagadougou	5	0.6%	97.8%
Kindi	4	0.5%	98.3%
Other	13	1.7%	100.0%

SARI – severe acute respiratory infection

*Boussé, Niou, Sigle, Sourgoubila, and Laye comprised the Boussé district hospital SARI Catchment Area as the cumulative number of proxy SARI diagnoses accounted for approximately ~ 85% of suspect SARI cases

†Number included (n = 781) is the total suspected SARI cases identified (n = 887) minus cases with repeated hospitalizations (n = 2) or missing information on residence (n = 104).

### Characteristics of suspect SARI cases in 2015 and 2016

Males accounted for 54.8% of the suspected SARI cases identified among catchment area residents, and 38.1% of suspected cases were in children less than one year old. Median age of persons with suspected SARI was 5 years. Outcome as recorded on hospital logs indicated that 40 (6.3%) suspected cases resulted in death during hospitalization.

Among all suspected SARI cases, 63.4% of cases had pneumonia as their only SARI diagnosis on either admission or discharge (**Table 2**). Other common diagnoses included bronchitis, asthma, and bronchiolitis. Approximately one in six cases (16.2%) had more than one SARI diagnosis listed on either admission or discharge. More than 10% of cases did not have a suspected SARI diagnosis at admission, and approximately 30% did not have a suspected SARI diagnosis at discharge. Most (76.8%) suspected SARI cases also had one or more additional non-SARI admission or discharge diagnoses. Malaria was the most common additional diagnosis, with 46.2% of suspect SARI cases also having an admission or discharge diagnosis of malaria.

**Table 2:** Hospital diagnoses of persons with suspect severe acute respiratory infection (SARI)

Diagnoses	Number with diagnosis, N = 755	Percent with diagnosis
Pneumonia	479	63.4%
Bronchitis	91	12.1%
Asthma	22	2.9%
Bronchiolitis	19	2.5%
Tuberculosis	15	2.0%
Other respiratory infection	7	0.9%
More than 1 SARI diagnosis*	122	16.2%

SARI – severe acute respiratory infection

*Patients had any two or more of the above categories at either admission or discharge; of these, the most common diagnoses were pneumonia (n = 104) and asthma (n = 45).

### SARI incidence

The overall SARI incidence rate for the Boussé CMA catchment area was 136 suspected SARI cases per 100 000 population (95% CI = 108, 152) in 2015 and 266 suspected SARI cases per 100 000 population (95% CI = 236, 300) in 2016 (**Table 3**). The highest incidence of suspected SARI was observed in children less than one year of age. In 2015 and 2016, children in this age group had SARI incidence rates of 1111 per 100 000 population (95% CI = 1047, 1178) and 2425 per 100 000 population (95% CI = 2330, 2524), respectively. The next highest incidence rate was in adults 65 years and older, with 377 per 100 000 population (95% CI = 341, 417) in 2015 and 816 per 100 000 population (95% CI = 762, 874) in 2016, respectively.

**Table 3:** Severe acute respiratory infection (SARI) incidence by age group for Boussé District Hospital catchment population in 2015 and 2016

Age group (years)	Number of suspect SARI cases	Total population	Incidence rate per 100 000	95% confidence interval
**2015:**				
<1	87	7834	1111	1047, 1178
1 to <2	3	7416	40	30, 55
2 to <5	22	20 638	107	88, 129
5 to <15	19	56 650	34	24, 47
15 to <50	58	76 430	76	61, 95
50 to <65	19	12 337	154	132, 180
≥65 y	27	7166	377	341, 417
All ages*	256	188 471	136	115, 161
**2016:**				
<1	195	8041	2425	2330, 2524
1 to <2	6	7610	79	63, 98
2 to <5	55	22 520	244	215, 277
5 to <15	52	56 792	92	75, 112
15 to <50	109	78 432	139	118, 164
50 to <65	29	12 660	229	201, 261
≥65	60	7354	816	762, 874
All ages†	514	193 409	266	236, 300

*6 individuals with missing age data were included in the all ages incidence calculation.

†8 individuals with missing age data were included in the all ages incidence calculation.

## DISCUSSION

We successfully applied WHO-recommended methods and used existing data to define burden of hospitalizations for severe respiratory disease in a low-income district in sub-Saharan Africa. Defining the SARI catchment area using patient residence data allows us to understand the population which the Boussé CMA primarily serves and to calculate SARI incidence rates. Estimates of SARI burden from low-income countries may provide a better understanding of the impact of SARI on vulnerable populations. While all age groups in our study were affected by SARI, the greatest burden was observed for children less than one year and adults 65 years of age or older for 2015 and 2016. These findings are consistent with other studies using comparable methodologies [4,13,14]. A study in Guatemala and Kenya found similar SARI incidence rates overall and for young children [15,16]. Incidence rates among those 65 and older were higher in our study than in Kenya. Thailand and Finland reported lower overall rates [4,13], but both countries are higher income level and have much better health care infrastructure; thus, lower incidence rates are not unexpected when compared with Burkina Faso. Both studies also included additional clinical information which was unavailable in our study.

SARI incidence rates in Boussé differed between years 2015 and 2016, overall, as well as by age group. The number of suspected SARI cases recorded from the Boussé CMA catchment area more than doubled between 2015 and 2016 (256 suspected cases in 2015 vs 514 suspected cases in 2016). There are several possible reasons why we observed this difference. First, differences in SARI rates could indicate a more severe season of acute respiratory illnesses in 2016 compared to 2015 for our catchment population, as the amount of disease caused by particular respiratory pathogens can vary from year to year. Second, we also observed that the total number of hospitalizations more than doubled in 2016 compared to 2015 (997 in 2015 vs 2037 in 2016), while the underlying population numbers for the Boussé CMA catchment area remained relatively the same (188 471 population in 2015 vs 193 409 population in 2016) [12]. One possible explanation for the increase in hospitalizations is that the government began offering free health care for children less than five years of age and pregnant women beginning in April 2016. With the introduction of free services, it is likely that more people sought care when they were ill. A study done in Burkina Faso in 2008 where free health care was piloted in one district showed that after introduction of free health care services, one out of every two illness episodes resulted in a health care facility visit compared to one in three episodes prior to the intervention [17]. An increase in the number of people aged 5-15 and the elderly indicates that more people overall from the catchment area came to the hospital in 2016 when compared to 2015. Finally, two SARI surveillance-related trainings were conducted for Boussé CMA staff between November and December 2016. These trainings may have led to better record keeping of SARI at Boussé CMA at the end of 2016 and a higher number of cases being recorded in the hospital logs. The months of November and December had a higher number of hospitalizations than most other months in 2016 and the highest number of SARI cases from 2015 and 2016 supporting the idea that record keeping improved. However, this is also the same time when SARI cases begin to increase and without comparable data from several prior years, it is difficult to confirm this hypothesis.

Our findings also highlight the presence of other diagnoses among patients with acute respiratory infections, with more than 75% of the cases in our study having an additional non-respiratory diagnosis in addition to the ARI diagnosis and nearly half having malaria. When estimating burden of SARI, it is important to include patients with multiple diagnoses who may not otherwise be included. This finding also indicates individuals are often quite ill, with multiple diseases on presentation to the district hospital.

There are some challenges when applying the WHO method. Many hospitals still rely on log books making data collection very time consuming. We employed a dedicated staff member to collect data rather than relying on existing facility staff to gather information. Additionally, we found that log books were often missing, in poor condition, or had illegible hand writing. Some of the missing log books were found in storage although this took time and delayed data collection. We also found that key information was often missing including residence information and discharge diagnosis information. To overcome these challenges, we assumed distribution of residence was the same for missing and non-missing patients. To address the problem with discharge diagnoses missing, we included both admission and discharge diagnosis. In some cases, we were able to refer to clinic notes to ascertain missing information. We also found there was variability in clinician diagnosing practices from the list of diagnoses to include from the WHO manual. We consulted clinicians at the hospital to understand under what circumstances they gave a diagnosis to ensure we were including the correct patient population. Some diagnoses we included were not listed in the WHO manual, but the clinical team at the hospital felt they should be included to capture all SARI cases.

There are some limitations to our study. The missing pediatric log books may have led to inaccurate estimations of total SARI cases in 2015 although we conducted single imputation to try to account for the missing data. Additionally, there are likely many people with SARI that never present to the hospital because of challenges with transportation to the hospital, ability to pay for services, or illness severity. In 2001, a study found that less than 22 out of 100 people accessed any services at a health facility at least once in a year [18]. This number may be even lower for older individuals or those who lack of financial resources and are not eligible for free health care or those who face significant challenges with transportation to the health care facility. Quantifying the number of people not accessing health care is difficult. For some cases, we were unable to determine if the respiratory diagnosis was chronic or acute due to lack of proper record keeping. Data for this study were drawn from Boussé CMA consultation and hospitalization log books, which contained only limited patient information. Due to missing and incomplete patient medical records, limited information from the hospital log books was available on the patients’ symptoms or clinical course. Over 15% of cases did not have any data on discharge diagnosis so we had to rely on a combination of information from both admission and discharge, and admission diagnoses are often inaccurate. Relying on Boussé CMA log books for diagnoses can be challenging due to differences in clinician diagnosing practices and missing data. Challenges with missing data and relying on admission diagnoses could have led to either over or under estimation of cases.

In spite of these limitations, our methods highlight how data readily available at district hospitals can be used to provide information useful for health policy. Local SARI burden estimates contribute to evidence-based decisions for local governments to consider when allocating scarce resources and planning intervention strategies to limit the spread of respiratory pathogens. These data may also be useful at the national level to compare to other districts with similar populations. Additionally, our data may be used to help establish baseline rates of SARI during various seasons which can be used to help identify respiratory disease outbreaks within the district and assess interventions.

## CONCLUSION

Our study estimated the catchment area and population for the Boussé district hospital utilizing the WHO method, and we were able to determine SARI incidence rates using data from hospital log books. Acute respiratory infections are a significant cause of disease burden among Boussé residents, particularly in children less than one year of age and in adults 65 years of age and older. Moving forward, the Ministry of Health will be able to utilize SARI surveillance data and the information collected in this study to monitor SARI incidence rates within the Boussé CMA catchment area and may consider applying a similar method at the national level. This surveillance system will better inform decision makers on the number of SARI cases, seasonality, morbidity and mortality, and specific pathogens circulating among this population. Additional data will support policy decisions to allocate resources and implement targeted interventions to address needs of those at high risk for respiratory infection.
